# Facile Control over the Supramolecular Ordering of Self-assembled Peptide Scaffolds by Simultaneous Assembly with a Polysacharride

**DOI:** 10.1038/s41598-017-04643-3

**Published:** 2017-07-06

**Authors:** Rui Li, Mitchell Boyd-Moss, Benjamin Long, Anne Martel, Andrew Parnell, Andrew J. C. Dennison, Colin J. Barrow, David R. Nisbet, Richard J. Williams

**Affiliations:** 1 School of Engineering, RMIT University, Melbourne, Victoria Australia; 2 Laboratory of Advanced Biomaterials, Research School of Engineering, The Australian National University, Canberra, ACT Australia; 3 Center for Chemistry and Biotechnology, Deakin University, Waurn Ponds, VIC Australia; 4 Department of Physics and Astronomy, University of Sheffield, Sheffield, United Kingdom; 5 Institut Laue Langevin, Grenoble, France; 6 Department of Chemistry, Technical University Berlin, 10623 Berlin, Germany; 7 School of Applied and Biomedical Sciences, Federation University, Mount Helen, Victoria Australia; 8 Biofab3D, St. Vincents Hospital, Melbourne, Victoria Australia

## Abstract

Enabling control over macromolecular ordering and the spatial distribution of structures formed via the mechanisms of molecular self-assembly is a challenge that could yield a range of new functional materials. In particular, using the self-assembly of minimalist peptides, to drive the incorporation of large complex molecules will allow a functionalization strategy for the next generation of biomaterial engineering. Here, for the first time, we show that co-assembly with increasing concentrations of a highly charged polysaccharide, fucoidan, the microscale ordering of Fmoc-FRGDF peptide fibrils and subsequent mechanical properties of the resultant hydrogel can be easily and effectively manipulated without disruption to the nanofibrillar structure of the assembly.

## Introduction

Molecular self-assembly is a process in which molecules interact with each other to spontaneously self-organise into higher order structures without external control^1^. This has been utilised as a versatile and accessible method to form complex functional materials^2–5^. Biology is replete with examples of complex structures that spontaneously form from seemingly simple starting materials^6, 7^. The self-assembly of amphiphilic peptides is based on the relative stability of the structures under the specific assembly condition (i.e. pH, temperature, ionic strength etc.)^8, 9^. Control over the organisation of the structures formed by self-assembling peptide (SAP) hydrogel systems are currently achieved via control of assembly conditions, such as the molecule’s specific pKa^10^, ionic strength^11^, chemical properties^12^, thermally induced gelation^13^, or biocatalytic control over the molecular interactions^14^. Typically, these systems consist of a single gelator species in a relatively dilute environment. The assembly of small molecules in the presence of other macromolecules such as polysaccharides can significantly alter the physical and mechanical properties of the final material by forming a complex with the gelators or the supramolecular architecture^15, 16^. Recently, the supramolecular ordering of self-assembled structures has been shown to be significantly altered when assembled in the presence of serum^17^ and cytosol proteins^18^. Control of such phenomena is critical for their application in medicine, as SAPs must assemble at physiological pH and in the presence of serum proteins. Recent studies of multiple component systems have shown SAPs to be an effective delivery tool for protein therapeutics^19^, viral particles^20^, cells^21^ and polyphenols^22^, which highlights the possibility of achieving supramolecular ordering in the physiological environment. Furthermore, we have recently achieved the *in vivo* presentation of biologically relevant basement membrane proteins^23^.

The incorporation of the gelling oligosaccharide agarose into a solution of low molecular weight gelators (supramolecular hydrogels) significantly improved the material properties of the hydrogel^24^. In order to study the possibility of including a functional polysaccharide into the peptide hydrogel, we chose to utilise a polyanionic biopolymer, fucoidan^25^. Fucoidans are vegetal fucose-containing polysaccharides extracted from brown algae and have low anti-coagulant activity^26^. They are biologically useful, as they are known to bind heparin-binding growth factors, and enhance their activity^27, 28^, whilst also being used for hemopoetic expansion culture^29^ and as wound healing accelerators^30, 31^. However, unlike agarose, fucoidan does not form gels in isolation, and requires gelling components to be added for wound care applications^31^. The fucoidan polysaccharide can therefore be considered as a polyelectrolyte, which we have demonstrated at low concentrations interacts with the supramolecular bundles and is presented on the surface of the fibrillar scaffold in a bioactive fashion without altering the scaffold^32^.

In this study, we set out to explore the ability of increasing the fucoidan concentration in solution as a facile method to modulate the assembly and mechanical properties of the resultant hydrogel through both binding and templating effects as the fibrils grew throughout the polyelectrolyte solution. This would provide proof-of-principle of a versatile technique of hydrogel formation from peptide-based gelators to not only distribute a variety of charged, functional biopolymers, within a gel matrix, as opposed to chemical modification and/or physical loading after gel formation, but also manipulate the properties of that scaffold. We chose a small, amphiphillic Fluorenylmethoxycarbonyl (Fmoc) pentapeptide that contains the biochemically useful peptide sequence Arginine-Glycine-Aspartic acid (RGD). Firstly, we examined the ability of this system to bind concentrations of fucoidan up to 10 mg/mL (0.1 wt%) under physiologically relevant conditions (pH 7.4 and 100 mM PBS concentration). We then determined the influence of the fucoidan on the assembly process and supramolecular ordering; finally we explored the effect of this process on the mechanical properties of the resultant hydrogel matrix.

## Results

### Initiation of Self-Assembly

**Figure 1:**
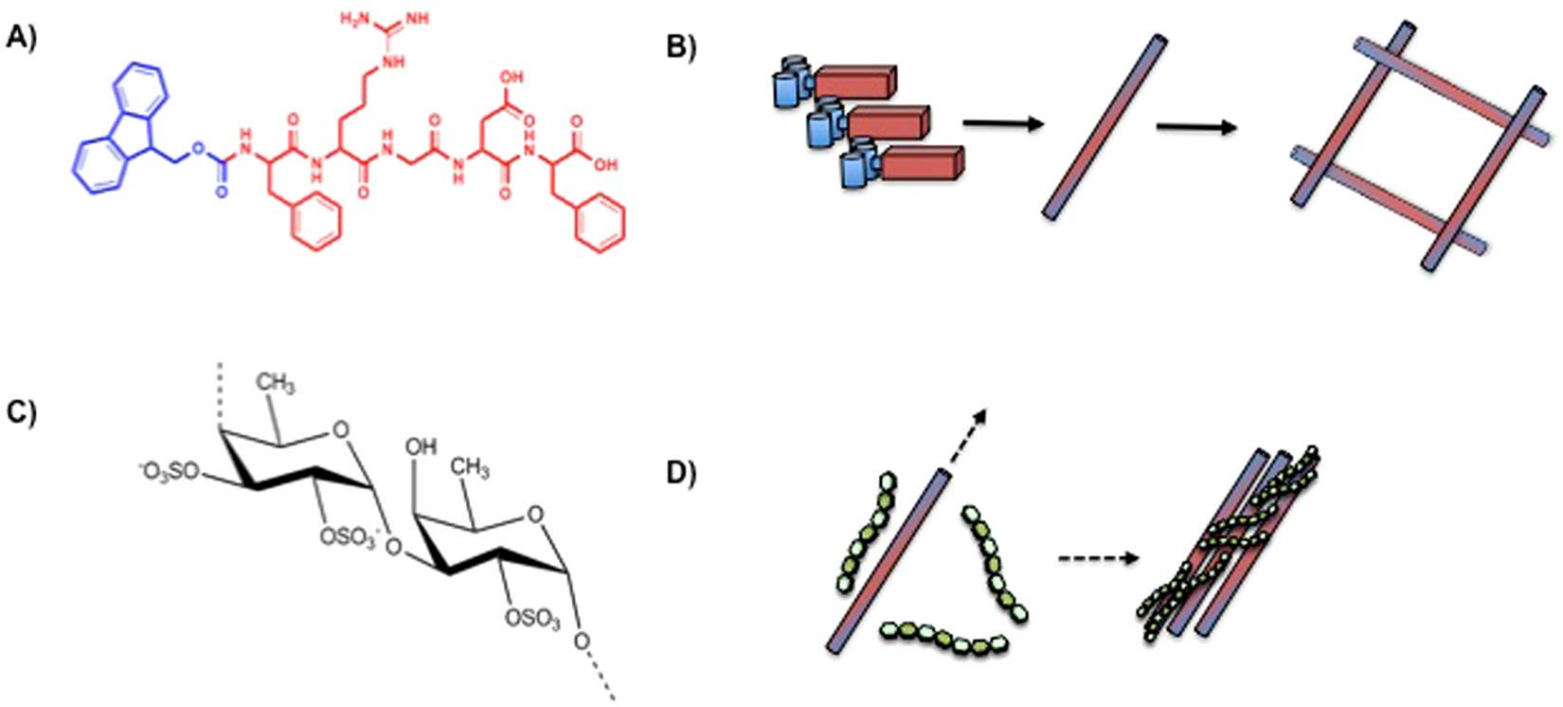
Peptide self-assembly: (**A**) structure of Fmoc-FRGDF. (**B**) schematic of the assembly process where individual peptides form fibrils which become entangled to form the hydrogel matrix. (**C**) repeating unit of fucoidan (**D**) Schematic of proposed induction of supramolecular ordering by interaction between fucoidan and individual fibrils to yield thicker bundles.

The underlying self-assembling system that was chosen for this study is based on amphiphilic aromatic peptide derivative, Fmoc-FRGDF (Fig. 1), as the mechanical properties of low molecular weight gelators of this class are difficult to modify^[2]^. These were prepared via solid phase Fmoc peptide synthesis methodology, which results in a white crystalline powder. Fucoidan was supplied as a highly soluble depyrogenated white powder. We examined four samples; a ‘pure’ hydrogel of Fmoc-FRGDF at 10 mg/mL, and three of the same concentration of peptide with +2, +5 and +10 mg/mL of fucoidan. In addition, a control was prepared where the gel was formulated from Fmoc-FRGDF and 10 mg/mL fucoidan was subsequently added by mixing. The hydrogels were prepared by a well-established pH switch methodology based on a Debye screening effect^10^. The peptide and/or fucoidan powders were solubilised by the addition of a small amount of NaOH to raise the pH. With vortexing, the pH was adjusted to 7.4 and buffered with potassium phosphate buffer. Hydrogelation was observed to have occurred within 2 hours of the process. Interestingly, the fucoidan/peptide gels were observed to assemble much more rapidly, possibly due to the interaction of the fucoidan with the individual peptide fibers as the hydrogel matrix was formed. This increase in gel formation has been previously observed as a function of the extent of the stimulus during the early stages of assembly^33, 34^. Importantly, the addition of the fucoidan did not affect the pKa of the peptide, and the gels all formed at the same pH^10^. A significant change in the apparent pKa of the SAP can occur, resulting in assembly at a pH value several units away from the calculated value^15^. Whilst the gels were all optically transparent, it was observed that the 10 mg/mL hydrogel was slightly more opaque (Figure S1A).

### Microscopic Analysis of Structure

**Figure 2:**
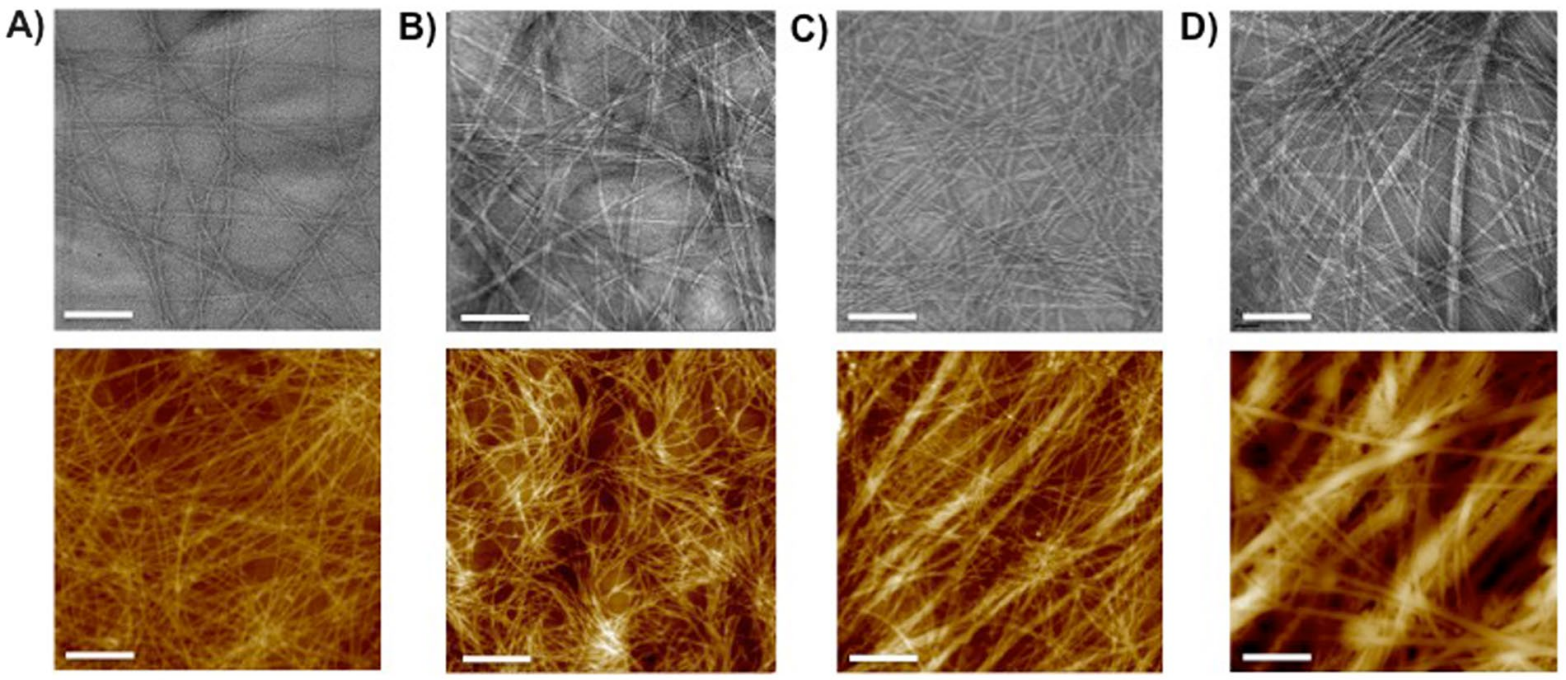
Nano- and microstructure of hydrogels (top panel TEM and bottom panel AFM): (**A**) 10 mg/mL Fmoc-FRGDF shows individual fibrils randomly interacting to form a matrix; (**B**) +2 mg/mL shows a small increase in thicker bundles where individual fibrils have aligned (**C**) +5 mg/mL and (**D**) +10 mg/mL fucoidan show a large number of thick bundles and respectively fewer individual fibrils. TEM scale bar represents 50 nm, AFM 1 µm.

In order to look at the effect of the fucoidan addition on the nano- and microstructure of the resultant hydrogels, we used transmission electron microscopy (TEM), atomic force microscopy (AFM), and small-angle neutron scattering (SANS). The TEM images show that the peptide alone forms a series of nanoscale fibrillar structures, ~10 nm in diameter and microns in length. Examination with AFM, revealed that these interact to form an interconnected network of individual fibrous structures ~50 nm in diameter (Fig. 2A). Importantly, with an increase in the concentration of fucoidan, the nanoscale self-assembled fibrils remained broadly consistent in morphology, with some increase in diameter seen at higher concentrations (Fig. 2B,D, top panel), while the microscale network organisation was observed to change significantly (Fig. 2B,D, lower panel). Here, we observed large, highly aligned ‘bundles’. +2 mg/ml resulted in no significant increase in diameter, whereas +5 mg/ml had a bundle width of ~50–100 nm. +10 mg/ml showed not only these large bundles of ~150 nm, but also alignment of these bundles into 1μm sized aligned features indicating that the fucoidan had an effect on the interactions between the fibrils at both the nano- and the microscale. It is likely that these large, almost macromolecular structures contribute to the opacity of the final hydrogel at this concentration A similar effect has been observed where Fmoc-dipeptide assembly was triggered via biocatalytic induction^35^, or using increasing ionic strength^11^. However, here we report the first example where a polyanionic polysaccharide has been ultilised to achieve increased supramolecular ordering using the pH switch methodology under biologically relevant conditions. When the control sample was analysed, thinner, and more curved fibrous structures co-existed with artefacts which are possibly ‘free’ fucoidan aggregates arising from the drying process closely associated with the surface of individual fibrils (Figure S1B). This was an interesting result, suggesting the polysaccharides had an affinity to, and were closely associated with, the fibrils. However, this was not sufficient to induce bundling during the peptide fibril assembly process.

### Small Angle Neutron Scattering for Analysis of the Interaction Between Fibrils and Fucoidan

**Figure 3:**
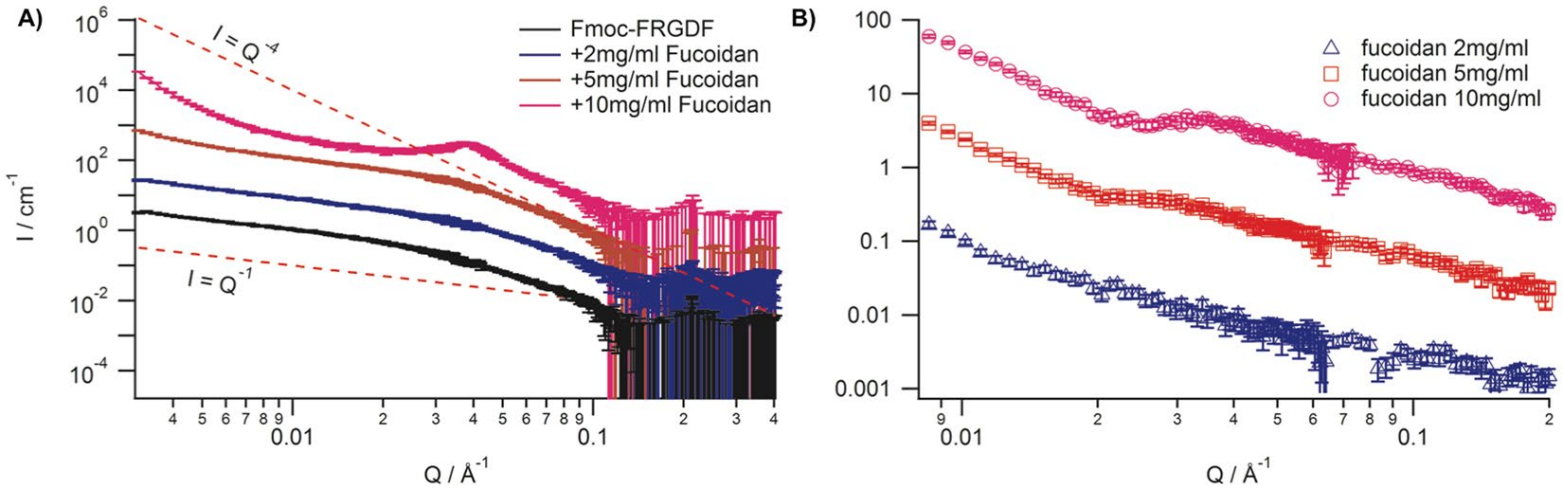
(**A**) SANS from hydrogels formed with fucoidan under conditions of fucoidan contrast matching, where increases in low Q scattering with higher fucoidan concentration result from an increased density of large objects in the sample. (**B**) SANS from pure fucoidan solution in D_2_O shows polyelectrolyte scattering with a single broad peak Q*.

To measure the gel structure without the added complication of drying/staining small angle neutron scattering (SANS) measurements were performed for the concentration series as well as for pure fucoidan (Fig. 3B). The scattering for pure fucoidan (Fig. 3B, left) was extremely weak even in pure D_2_O solution due in part to the large amount of exchangeable hydrogen atoms as well as the low concentration of fucoidan. The polysaccharide in isolation showed concentration dependent behaviour similar to that observed for other polyelectrolytes^36, 37^ with broad correlation peaks apparent for both the 5 mg/mL and 10 mg/mL concentrations and exhibiting low Q scattering giving ${\boldsymbol{I}}({\boldsymbol{Q}})\propto {{\boldsymbol{Q}}}^{-2.7}$ scaling for 5 mg/mL and ${\boldsymbol{I}}({\boldsymbol{Q}})\propto {{\boldsymbol{Q}}}^{-3}$ for 10 mg/mL and correlation peaks centred at Q^*^_5_ = 0.028 and Q^*^_10_ = 0.032, which we interpret as a network having mesh sizes of d^*^_5_ = 22.4 nm and d^*^_10_ = 9.8 nm. This presence of a mesh structure in solution prior to the initiation of peptide assembly potentially explains some of the morphological differences observed between hydrogels formed in the presence of fucoidan and those where fucoidan was added after assembly as the fucoidan conformation could be expected to differ greatly. The Debye screening effect of the pH switch used to initiate assembly and then subsequent pH adjustment means that the final ionic strength of the hydrogels was around 0.1 M and that this was sufficient for the fucoidan polymer to have random coil conformation^38^. In addition, we expect the emerging SAP fibrils that had an affinity for fucoidan, would influence polymer conformation. Qualitatively, the SANS measurements of the peptide hydrogels showed trends in agreement with the *ex-situ* TEM and AFM characterisation (Fig. 3A). We interpret the increases in low Q scattering with higher fucoidan concentration to result from an increased density of large objects in the sample whose size was too large to be resolved with the available Q range. SANS from the hydrogels formed by peptide assembly in the presence and absence of fucoidan were performed under two conditions; at 21.5% D_2_O, where fucoidan was rendered invisible through isotopic contrast matching and in pure D_2_O where all components of the hydrogel should have sufficient contrast to contribute to the scattering. The scattering under conditions of 21.5% D_2_O for the fucoidan-free, 1 mg/mL, and 5 mg/mL conditions displayed rod-like scattering objects with intermediate Q scattering proportional to Q^−1^ crossing over to Q^−4^ at a scattering vector corresponding to the fibril radius The data for the highest fucoidan concentration of 10 mg/mL, presenting strong differences in the scattering also follows this trend although additional contributions to the scattering were present. Model independent analysis showed that initially there was a subtle thinning of the fibril radius upon addition of fucoidan from 4.9 ± 0.1 nm to 3.6 ± 0.1 nm. Analysis of the intermediate Q region appeared to indicate that the average structure of the individual SAP fibrils remains constant across the range of fucoidan concentrations studied. SANS from the peptide assemblies at the two higher fucoidan concentrations exhibit greater structural differences with both samples showing increased scattering at low Q with rising fucoidan concentration which can be interpreted as increasingly large scattering objects within the hydrogel.

Fitting different Q-ranges for each of these data with power laws highlights the different contributions to the scattering in each case. Increasing to 5 mg/mL fucoidan an increase in intensity at low Q was visible and a clear deviation from I α Q^−1^ scattering to a Q^−1^ ^15^. power law which we interpret to be due to dilute large aggregates in the hydrogel. This rise in low Q scattering intensity was much more pronounced in the highest fucoidan concentration and appears to follow a Q^−4^ decline which we interpret to be Porod scattering from an increased concentration of large bundles. Due to the limited Q-range of the measurement we were unable to estimate average lateral bundle sizes. However, the presence of a Bragg peak at Q = 0.0376 Å^−1^ indicates the presence of a characteristic length-scale of 16.7 nm. As this was approximately twice the fibril radius we observe in solution correlations, we interpret this peak as a lateral correlation between two individual fibrils within in the bundles when hydrated, as was observed in the dried samples observed by microscopy.

### Analysis of Molecular Ordering Within the Fibrils

**Figure 4:**
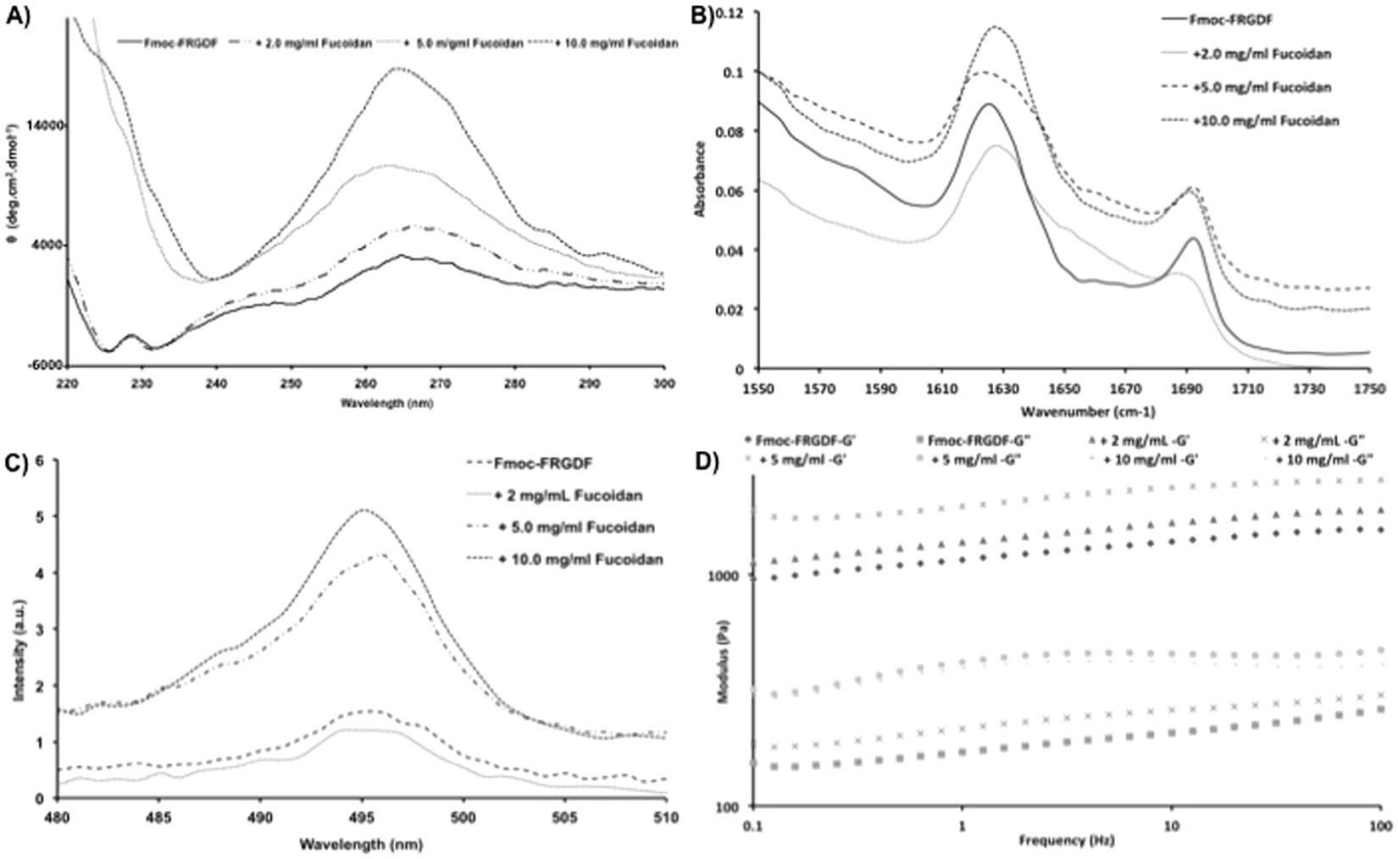
Spectroscopic analysis of peptide assembly with increasing concentrations of fucoidan: (**A**) CD spectra showing increased magnitude in the region located at 260–270 nm associated with increasing supramolecular organisation; (**B**) FT-IR spectra showing consistent β-sheet formation across all conditions suggesting the assembly mechanism of the peptide is not disrupted by increasing concentrations of fucoidan (**C**) Fluorescence spectra of the region 480–510 nm showing increasing fluorescence from Fmoc ordering into a consistent dominant aggregate. (**D**) Rheological Characterisation of the hydrogels. Typical frequency sweeps showing elastic (G’) and loss (G”) moduli, where the increase in fucoidan is associated with a increase in stiffness.

A number of spectroscopic analyses were used to determine if the addition of the fucoidan to the peptide system maintained the molecular organisation within the fibrils observed via TEM, as opposed to promoting a different organisation^17^. Fourier transform infra-red spectroscopy (FTIR) was used to analyse the vibrations within the amide I region (1600–1700 cm^−1^). These are typically used to determine the nature of the secondary structure formed between peptide sequences. Analysis of all the systems revealed conserved features; a major peak at approximately 1690 cm^−1^ and a minor peak at 1630 cm^−1^ (Fig. 4B), indicating the presence of β-sheet interactions and a carbamate typical of this class of assembly. The inter- and intrafibrillar excitonic interactions that arise from the assembly process have been shown to give rise to pronounced chiral spectra when analysed with circular dichroism (CD)^10, 23, 33^. Fmoc-peptides are chiral molecules, which, when hierarchically organised into self-assembled structures produce pronounced CD signals ^39^. CD analysis of the π-β class of assembled fibrils maintains characteristic transitions over three distinct regions (Fig. 4B). Those in the 300–310 nm region are the result of aromatic Fmoc- orientating via π-stacking, in accordance with other similar assemblies^10, 40^; however, the full transitions in the amide region of the system (below 220 nm) that indicates the presence of β-sheets, were affected by scattering in the higher concentrations of fucoidan^40^. Finally, the region between 230–270 nm represented bundling between the fibrils driven by surface interaction signals, analogous to large macromolecules^14, 33^. Importantly, when the control of post assembly fucoidan addition was prepared, the spectra were unchanged from the Fmoc-FRGDF spectra, indicating that the effect of supramolecular bundling occurs during the self assembly process (Figure S2A). The extensive interactions between the Fmoc- groups gives rise to significant fluorescence emissions. The fluorescence emission spectra of the hydrogels formed all showed characteristic features expected from this class of materials^40^ (Figure S2B). The monomeric peak centered on 310 nm was observed to increase with the addition of fucoidan up until 10 mg/mL, whereupon it decreased. This is likely due to the slight opacity of this gel leading to quenching effects^14^. A broad peak centred on 495 nm represents excimer species^40^. This peak is shown in Fig. 4C, and can be associated with the formation of an extended J-aggregate^14^. This feature increased in intensity in the order of Fmoc-FRGDF<+2<+5<+10. This can be interpreted as increased supramolecular ordering arising from the interactions of the fucoidan with the fibrillar structures^33^. Again, when the control was compared, the magnitude of the emission in this region was significantly less, indicating that no supramolecular ordering was observed when fucoidan is added post-assembly (Figure S2C).

### Mechanical Testing to Evaluate the Effect of Increased Bundling

Lastly, to explore the effect on mechanical properties that arise due to increased supramolecular ordering, oscillatory rheology was preformed (Fig. 4D). The characteristic frequency sweeps of all four hydrogels showed that each system forms a hydrogel where the elastic modulus (G’) dominates the loss moduli (G”) over a range of frequencies. As expected a partial frequency dependence was observed and a thickening effect occurred at high frequencies due to water loss typical of non-covalent entangled networks^23, 41, 42^. The hydrogel formed from Fmoc-FRGDF alone was weak (G’ ~10 Pa), this increased by several orders of magnitude to ~1500 Pa with the addition of 1 mg/mL of fucoidan. Separate analysis of the fucoidan solution showed that it contributed no significant value to the system via a separate gel contribution (Figure S2D); therefore, the increase in elastic moduli directly results from interactions between the fucoidan and the peptide fibrils. As the concentration of the fucoidan increased, the elastic moduli stabilised at ~2000 Pa. We believe that this is due to the increased strength of the thicker bundles being offset by a reduction in entanglements, which resulted from the formation of solitary fibrils.

## Conclusions

This work shows that the assembly of Fmoc-peptides can be influenced by the addition of polysaccharides, producing cooperative effects that, whilst not competing with the kinetically stable individual fibrils, lead to markedly different ordering of the resultant structures. This is the first example of the use of a polysaccharide at room temperature and at physiological pH and ionic strength to modify the alignment and mechanical properties of the resultant matrix. In addition, it shows that by using the pH switch methodology similar effects can be achieved as those produced using enzymes. Therefore, the pH switch methodology represents a simpler route to access these final structures, without having any potentially unwanted residual biocatalysts upon application. A number of biologically important macromolecules carry surface charge, and are able to act as a polyelectrolyte in solution. We demonstrate a simple method to functionalised nanomaterials, by initiating self-assembly within a solution of polysaccharide. Importantly, this process allows the nanostructured fibrils to form whilst interacting with the charged polymer in solution, in a concentration dependant fashion without significantly altering the internal ordering, but having a significant effect on the final material. We believe that this approach will allow the facile production of nanostructured, multicomponent biomedical materials, in particular for the incorporation and presentation of other biologically functional macromolecules.

## Methods

### Peptide synthesis

Peptides were synthesised using a standard Fmoc chemistry procedure on a 0.4 mmol scale in a glass reaction vessel with sintered glass filter. Briefly, the Fmoc-Phe-wang resin (GL Biochem, Shanghai, China) was swelled in dehydrated N,N-dimethylformamide (DMF) for 15 minutes, then the reaction vessel was drained and the resin was washed with more DMF. 10 mL 20% piperidine in DMF was added to the resin for the deprotection of the amine terminus by rotating the reaction vessel for 15 minutes, then draining the reaction vessel and washing with DMF. A Kaiser test was performed to confirm the presence of free amine groups after washing away residual DMF with dichloromethane (DCM), and then the resin was washed again with DMF. In the coupling stage, 2.0 mmol Fmoc-amino acid, 1.9 mmol 2-(1H-Benzotriazole-1-yl)-1,1,3,3-Tetramethyluronium hexafluorophosphate (HBTU), 2.0 mmol 1-hydroxybenzotriazole hydrate (HOBt), and 4.8 mmol N,N-diisopropylethylamine (DIPEA) were added to a glass vial and dissolved in 8 mL DMF. The solution was pre-activated by shaking the vial violently for 2 minutes, and then this reactant was transferred into the reaction vessel and reacted for 40 minutes. After coupling, the reaction vessel was drained and the resin washed with DMF followed by another Kaiser test to confirm the absence of free amine groups. After completion of the peptide sequence by repetition of the above steps, the resin was washed with ethanol and transferred to a glass vial, then dried under vacuum overnight. The peptide was cleaved from the resin with a cocktail of 95% TFA, 2.5% TES and 2.5% water, followed by precipitation in dry ice cooled diethyl ether. The white precipitate was collected and dried under vacuum for 24 hours before use. Reverse phase high performance liquid chromatography (RP-HPLC) was used to determine the purity of the desired sample. mp: 189.9–191.2 °C; ^1^H-NMR (500 MHz, D_2_O/DO^−^): δ 7.83–7.23 (m, 10H, Phe_1_, Phe_5_), 6.90 (bs, 2H, Fmoc), 6.83 (bs, 2H, Fmoc), 6.62 (bs, 2H, Fmoc), 6.57 (bs, 2H, Fmoc), 5.20 (bs, 2H, Fmoc), 4.58 (dd, *J* = 5.6, 5.0 Hz, 1H, Asp_2_), 4.42 (dd, *J* = 7.7, 5.2 Hz, 1 H, Phe_1_), 4.32 (d, *J* = 7.1, 7.1 Hz, 1H, Phe_5_), 4.27 (dd, *J* = 8.6, 5.4 Hz, 1H Arg_4_), 3.86 (d, *J* = 16.9 Hz, 1H, Gly_3_), 3.81 (d, *J* = 16.9 Hz, 1 H, Gly_3_), 3.15 (dd, *J* = 14.2, 5.5 Hz, 1H, Phe_1_), 3.12 (t, *J* = 6.8, 1H, Fmoc), 3.05 (dd, *J* = 13.7, 7.1, 1H, Phe_5_), 2.98 (dd, *J* = 13.7, 7.1, 1H, Phe_5_), 2.97 (dd, *J* = 14.2, 5.5 Hz, 1H, Phe_1_), 2.62 (dd, *J* = 16.1, 4.7 Hz, 1H, Asp_2_), 2.46 (dd, *J* = 16.0, 8.7 Hz, 1H, Asp_2_), 1.82 (m, 2H, Arg_4_), 1.67 (m, 2H, Arg_4_), 1.51 (m, 2H, Arg_4_); ^13^C-NMR (125 MHz, CDCl_3_, from HMBC and HSQC data): δ 177.8, 177.6, 175.7, 173.67, 172.1, 170.5, 163.3, 156.8, 139.7, 137.3, 137.2, 136.8, 129.3, 129.2(5), 128.7,128.6, 127.9, 126.8, 126.7, 126.3, 120.4, 119.1, 107.3, 57.0, 56.2, 52.9, 51.2, 42.1, 40.2, 38.1, 37.5, 37.3, 27.9(3), 27.8(8), 24.0; HRMS (*m/z*): [M+H]^+^ calcd for [C_45_H_50_N_8_O_10+_H]^+^, 863.37227; found, 863.37290.

### Hydrogel formation

10.0 mg of crystalline Fmoc-FRGDF along with mixtures of 1, 5, 10 mg fucoidan (Marinova Pty Ltd, Cambridge, Tasmanian, Austrlaia) were added to separate 4 mL glass vials. 400 µl Milli-Q water (purified by Milli-Q Advantage A10 System, Merck Milipore, Australia) was added into each vial, then pH was increased by the addition of a minimal volume of 0.5 M NaOH with vortexing until a transparent solution was obtained. The solution was then neutralised to pH 7.4 via drop wise addition of 0.1 M HCl (Asia Pacific Specialty Chemicals Ltd., Australia) with vortexing (to avoid precipitation). Finally, 100 mM PBS (pH 7.4) was added into the solution to bring the total volume up to 1.0 mL. The solutions were then kept undisturbed at room temperature for 24 hours to ensure gelation (total peptide concentration was 1 wt%).

### Circular dichroism

Hydrogel spectra were measured using a Jasco J-815 circular dichroism spectrometer with the spectral bandwidth 1 nm and integrations 2 s^−1^. A 1 mm quartz cell (Starna Pty. Ltd., Australia) was used. Samples were prepared at a concentration of 0.06%. The data were collected 3 times and average values were used for all the samples.

### Fourier transform infrared spectroscopy

A Nicolet 6700 fourier transform infrared spectroscopy (FT-IR) was used to collected spectra using attenuated total reflection (ATR) mode. 12 μL of hydrogels were applied directly to the ATR crystal and scanned between the wavenumbers of 4000 and 400 cm ^−1^ over 64 scans. A background scan of PBS buffer was applied before the samples.

### Fluorescence spectrophotometer

Fluorescence emission spectra were measured on a Cary Eclipse Fluorescence Spectrophotometer (Agilent Technologies, USA) with light measured orthogonally to the excitation light. The emission bandwidth was set at 5 nm. A scanning speed of 600 nm min^−1^ was used with a data pitch of 1.0 nm. Excitation wavelength was at 248 nm and emission data range between 300 nm and 600 nm. A quartz cuvette (Starna Pty. Ltd., Australia) of 1 mm path length was used for scanning. Samples were prepared at a concentration of 0.5 wt%.

### Transmission electron microscopy

JEOL-2100 LaB_6_ transmission electron microscopy (TEM) (JEOL Ltd., Japan) at an operation voltage of 100 kV was used for TEM images. Lacey carbon coated films (Agar) on 300 mesh copper grids (Emgrid Pty. Ltd., Australia) were used as sample holders. For sample preparation, 12 µL of sample was applied onto the grid and allowed it to absorb for 5 minute, then using split Whatman filter paper (No.1) to wick off excess fluid. One drop of negative stain NanoVan (Bio-Scientific Pty. Ltd., USA) was put onto parafilm “M”, then the grid was placed on the stain with carbon side down and allowed to stain for 5 minutes. Subsequently, the samples were dried in air for 2 minutes with carbon side up, at last the grids were placed into the grid box and left to dry overnight.

### Atomic force microscopy

Atomic force microscopy (AFM) images of the samples were obtained using a Multimode 8 (Bruker BioSciences Corporation, USA). For sample preparation, 15 μL hydrogel was applied on highly ordered pyrolytic graphite (HOPG) substrates (SPI, USA), the redundant samples were absorbed by pipette. The hydrogels formed with 0 and 1 mg/mL fucoidan were at a concentration of 0.05%. The tips used were scanasyst-air probes with silicon tip on nitride lever (Bruker BioSciences Corporation, USA). The AFM was operated in peak force QNM. Calibration of deflection sensitivity, spring constant and tip radius of probes was done before sample imaging. Scan size was fixed at 10 µm^2^.

### Small-angle neutron scattering

Small-angle neutron scattering (SANS) was performed using the D33 instrument at the Institut Laue-Langevin, Grenoble, France^43^. Scattering was measured at 2 sample-detector distances in fixed wavelength mode using a wavelength of 6 Å and a wavelength resolution of Δλ = 10% at detector distances of 2 m and 12 m to cover the Q-range 0.03–0.5 Å^−1^. Data collected for the two detector distances were reduced using the GRASP program^44^ and joined using the NIST SANS package for Igor Pro^45^. Hydrogel samples were prepared at 21.5% D_2_O in order to contrast match to fucoidan, leaving only scattering from the peptide assemblies and pure fucoidan was prepared in D_2_O solution for maximum contrast and reduced background. The samples were measured in sealed 1 mm path-length Hellma cells and after injection of these highly viscous hydrogels each sample was heated in a waterbath to 70 °C to remove shear-alignment from the sample loading.

### Rheology

A Discovery Hybrid Rheometers (TA Instruments, USA) was operated at constant stress with a strain of 2.83%. An amplitude sweep was performed and showed no variation in G’ and G” up to a strain of 60%. Frequency sweeps were performed over a range between 0.1 and 100 Hz. Temperature was maintained at 25 °C via the use of Peltier plate control. Hydrogels were performed on a cone-plate geometry (40 mm, 2 ° 1′ 37”) with a gap of 51 µm. A water trap was used to minimise evaporation.

## Electronic supplementary material

Supplementary Information
