# Supplementary material for: Facile Control over the Supramolecular Ordering of Self-assembled Peptide Scaffolds by Simultaneous Assembly with a Polysacharride
Source: Sci Rep. 2017 Jul 6;7:4797. doi: 10.1038/s41598-017-04643-3 (PMC5500548; doi:10.1038/s41598-017-04643-3)
Supplement: Supplementary file 1 — Supplementary Information [file 41598_2017_4643_MOESM1_ESM.doc]

**Supplementary Information**

**Facile Control over the Supramolecular Ordering of Self-assembled Peptide Scaffolds by simultaneous assembly with a Polysacharride**

Rui Li^3^, Mitchell Boyd-Moss^1^, Benjamin Long^3^ Anne Martel^5^, Andrew J. Parnell^4^, Andrew J.C. Dennison^4,5,6^ Colin J. Barrow^3^, David R. Nisbet^2#^ and Richard J. Williams^1#*^

^1^School of Engineering, RMIT University, Melbourne, Victoria, Australia

^2^Laboratory of Advanced Biomaterials, Research School of Engineering, The Australian National University, Canberra, ACT, Australia

^3^Center for Chemistry and Biotechnology, Deakin University, Waurn Ponds, VIC, Australia.

^4^ Department of Physics and Astronomy, University of Sheffield, United Kingdom

^5^Institut Laue Langevin, Grenoble, France

^6^Department of Chemistry, Technical University Berlin, 10623 Berlin, Germany


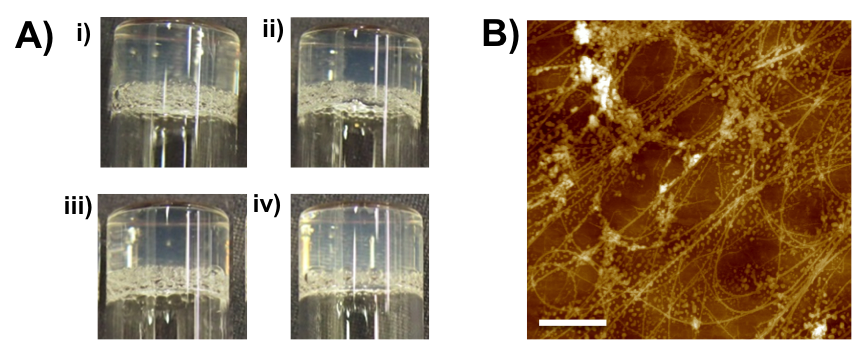


**Supplementary Figure 1: A) Gels formed from i) Fmoc-FRGDF ii) +2, iii) +5 and iv) +10 mg/mL fucoidan. B) AFM showing the lack of bundling and presence of clusters if fucoidan is added after the gel has formed.**


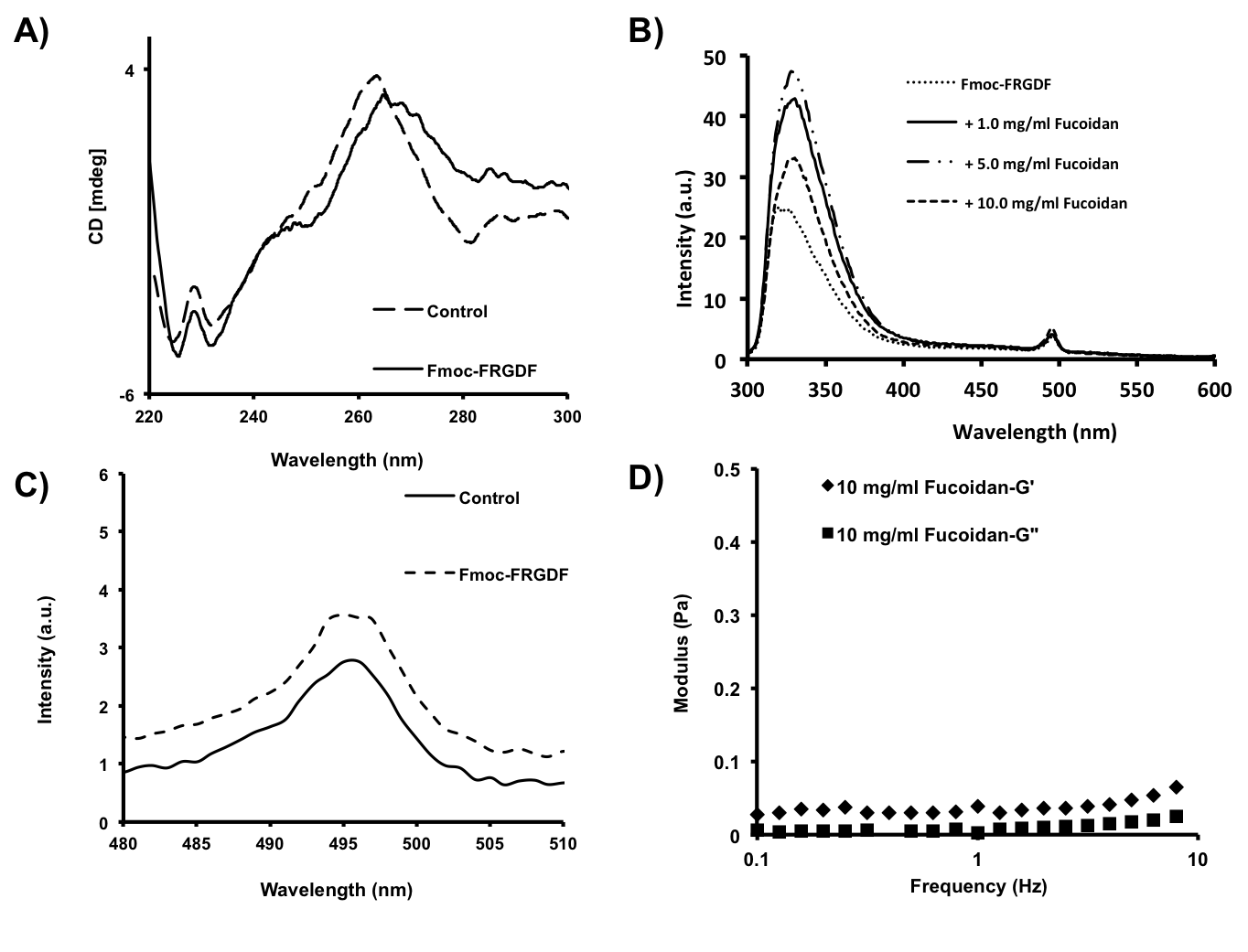


**Supplementary Figure 2: A) CD spectra for the control gel (fucoidan added post assembly) vs. Fmoc-FRGDF B) full fluorescence emission spectra for the samples C) Control vs Fmoc-FRGDF of the feature centered on 495nm D) Frequency sweep analysis of a solution of 10 mg/mL fucoidan showing no significant elastic contribution to the system.**
